# How talent management execution impacts career experiences: exploring the organization-individual intersect

**DOI:** 10.3389/fpsyg.2024.1272645

**Published:** 2024-01-26

**Authors:** Marna van der Merwe, Petrus Nel, Crystal Hoole

**Affiliations:** Department of Industrial Psychology and People Management, School of Management, College of Business and Economics, University of Johannesburg, Johannesburg, South Africa

**Keywords:** talent management, careers, career experiences, talent strategy, talent philosophy

## Abstract

The aim of the research was to investigate the ways in which talent management execution impacts the individual career experiences of talent, specifically exploring how talent management execution and career enablement can be closer aligned to unlock value for both the organization and the individual. A qualitative research design was employed to explore the research question. During the first phase of the research data was collected from 13 talent management professionals using purposeful sampling and semi-structured interviews. During the second phase of the research the Delphi method was used to validate the findings (*n* = 8). Four broad themes were identified as the most prominent ways in which talent management execution impacts the career experiences of individuals. The first is the philosophical underpinning that the organization holds in how talent is defined as well as the exclusivity or inclusivity of their approach. The second is the identification criteria used to identify talent within the organization. Thirdly, the extent to which talent management practices are aligned with career enablement practices and the congruence between these. Lastly, the role of the psychological contract and employee experience in managing mutual expectations. The resultant tension must be proactively managed by (1) clearly articulating the talent philosophy and making this visible through talent management execution, (2) ensuring alignment between talent management practices and the career enablement available to talent, and (3) delivering on expectations through the lived experiences of talent. The study contributes to the existing body of knowledge on talent management and careers, with a specific focus on the intersection between these and defining ways to align these practices to create congruent and authentic career experiences for individuals.

## 1 Introduction

The way organizations think about and manage talent is continuously challenged, as globalization, digitization and technological augmentation disrupt how work, workplaces and workforces are defined (Cascio and Aguinis, 2019; Claus, 2019). One of the most prominent changes is in the roles that organizations and employees play within the employment relationship, which have shifted expectations pertaining to the psychological contract and careers (Cascio and Aguinis, 2019).

Within this context, the nature and experience of careers have also evolved (Miś, 2018). Careers are increasingly self-managed, and the psychological contract has evolved to reflect the volatility, ambiguity and uncertainty of the organizational context (Miś, 2018). The role that the organization plays in career management has also changed over time, as careers become more boundaryless and independent of organizational structures (Wiernik and Kostal, 2019). This means that traditional talent management practices seemingly contradicting emerging contemporary career trends, as well as the evolution of the organizational career (Crowley-Henry et al., 2019; Wiernik and Kostal, 2019).

For talent management to be successful and enable organizational success, the complex nature of careers must be considered in the operationalization of talent management (Crowley-Henry et al., 2019). In particular, moving beyond understanding talent management as a stepwise and linear organizational process, to aligning talent management practices with the changing nature of careers, is critical in the changing world of work (Mensah, 2019).

Whilst some research within the talent management domain has linked career theory to talent management (Yildiz and Esmer, 2021), there is still largely a lack of comprehensive research on understanding how the careers ecosystem are linked to talent management practices (Crowley-Henry et al., 2019; Gallardo-Gallardo et al., 2020). Although talent management practices have a direct impact on individuals, the experiences of employees and their reaction to talent management in the context of their careers have largely been overlooked in talent management research (Gallardo-Gallardo et al., 2020; Malik and Singh, 2020).

The aim of the research was to investigate the ways in which talent management execution impacts the individual career experiences of talent, by exploring touchpoints and intersections between talent management practices and individual careers. More specifically, the study explores how talent management execution and career enablement can be closer aligned, to unlock value for both the organization and the individuals and, in turn, ensure a sustainable talent supply.

## 2 Theoretical framework

To frame the boundary of the study, the relevant theoretical underpinnings are discussed in this section. Firstly, the changing definition and scope of careers and the relevance of career sustainability and related career constructs in the context of talent management are outlined. Secondly, an overview of organizational talent philosophies as the philosophical underpinning of talent management execution is provided, which impacts careers. Lastly, an overview of the role of the psychological contract is provided, and its role in managing the tensions and mutual expectations that exist between individuals and the organization. This forms the theoretical foundation of the study.

### 2.1 Defining contemporary careers and its relevance

The concept of careers and its definition has shifted over time as the world of work has changed (Wiernik and Kostal, 2019). This has resulted in employees seeking more control in their careers, exercising more freedom and choice in how their careers are managed (Crowley-Henry et al., 2019; Rodrigues et al., 2020). Contemporary models of careers focus on career self-management, mobility and employability, independent of organizational constraints (Akkermans et al., 2018).

In the context of contemporary careers, internal values as well as self-direction are considered the most important drivers of career choice and decisions (Jo et al., 2023). In a recent meta-analysis study, very little evidence was found to distinguish protean and boundaryless careers as two separate career orientations (Wiernik and Kostal, 2019). Instead, the concept of career self-management and a proactive orientation to career ownership are central to these career orientations (Wiernik and Kostal, 2019). This self-directed nature of careers can lead to a disconnect between individuals and the organization, within which their careers are experienced. Through understanding the career concepts associated with traditional organizational, boundaryless and protean careers, talent management within the context of these career structures can better be explained, informed and adapted (Crowley-Henry et al., 2019) to enable and facilitate career sustainability.

Career sustainability is defined as “sequences of career experiences reflected through a variety of patterns of continuity over time, thereby crossing several social spaces, characterized by individual agency, herewith providing meaning to the individual” (Van der Heijden and De Vos, 2015, p. 7). Sustainable careers are characterized by aligning work with individual strengths, ongoing learning and opportunities for development, security via employability and enhanced marketability (internal and external) and work-life fit over time that provides adequate engagement in both domains (De Vos and Van der Heijden, 2017; McDonald et al., 2022). A sustainable career addresses both the needs of individuals and organizations, as well as the challenges associated with contemporary careers (De Vos et al., 2020). The attributes and characteristics of sustainable careers ensures that the core objectives of talent management efforts are met. For organizations, a focus on sustainable careers provides maximum return on investment by aligning individuals to where they add the most value, continuous enhancement of organizational competencies through ongoing learning and development, stability of the workforce through increased adaptability in change and organizational commitment and retention of talent through engagement (Valcour, 2015; McDonald et al., 2022). A detailed overview of the attributes of sustainable careers as well as the associated organizational alignment and expected benefits are outlined in Table 1.

**Table 1:** Overview of the attributes of sustainable careers as well as the associated organizational alignment and expected benefits.

Individual career sustainability attribute	Organizational talent management objectives	Expected outcomes for individuals and organizations
*Individual work alignment*	Maximum human capital yield	When employees do work that is aligned with their interests and strengths, organizations gain maximum benefit from their human resources. Employees generally feel more motivated, find meaning in their work and are generally more committed to the organization where they are doing the work. This has a direct positive impact on organizational outcomes such as increased productivity and retention of employees.
*Continuous individual learning*	Reskilling and updating of organizational competencies and skills	The ability of organizations to access and deploy the skills needed to deliver on its strategic intent is a competitive advantage and key to organizational success. Similarly, for individuals to remain relevant and sustain their careers, their skills have to be updated through continuous learning. In turn, having a workforce that is engaged in continuous learning, provides organizations with access to skills and evolves organizational capabilities over time.
*Increased job security for individuals through employability*	Stable talent supply and adaptability for organizations	Continuous learning and enhancing of skills increase individual employability of employees. Similarly, continuous learning and reskilling of employees within organizations creates stability for organizations and assists in adapting to external market changes and competitive pressures. Employees engaged in learning typically perform better and are in general more proactive in their careers. Organizations who have continuous learning systems in place typically perform better and are able to adapt to changes with more ease.
*Individual work–life fit over the lifecycle*	Retention and commitment	Sustainable careers are dynamic in nature and reflect the self-directed nature of contemporary careers. This means that employees strive to integrate and modify their work arrangements across their careers in order to balance other life demands. This means that organizations who place less emphasis on rigid, traditional work arrangements are able to avoid the career withdrawal often associated with conflicting work and life demands of employees. This mutual career flexibility is associated with increased retention and commitment from employees.

Adapted from Valcour (2015) and De Vos and Van der Heijden (2017).

This insight into sustainable careers provides the basis for understanding the dynamics and complexities of careers that must be considered within talent management, as well as the ways in which talent management can support and enable sustainable careers.

### 2.2 Organizational talent philosophies

Talent philosophies refer to the underlying beliefs that are held about talent, its interpretation, and ultimately the value and instrumentality that it holds in organizations (Bonneton et al., 2020). Therefore, talent philosophies affect the way that talent management is operationalized (Meyers et al., 2019). The value of talent philosophies resides in its ability to assist in managing the tensions that exist in the field of talent management (Meyers et al., 2019; Bonneton et al., 2020). This is predominantly focused on the tension that exist between inclusivity vs. exclusivity of talent, as well as the tension between innate or attained (developable) talent. These tensions are seen to have the biggest impact on how talent management is operationalized in organizations (Meyers et al., 2019; Sparrow, 2019; Vardi and Collings, 2023) and ultimately the experience of talent.

### 2.3 The role of the psychological contract – the talent management and careers intersect

The impact of contextual factors, such as the changing world of work and the nature of careers have a significant impact on the employer-employee exchange within the psychological contract (Raheem and Kahn, 2019; Coetzee and Deas, 2021). The biggest shift in the psychological contract is based on the mutual exchange between employees and employers, to ensure both career and organizational sustainability (Donald et al., 2019). The new psychological contract also involves multiple role players and relationships, with the increase in cross-boundary working and evolving portfolios of employment contracts, as opposed to a single employment contract over a period (Perkins et al., 2021). The psychological contract plays an important role in managing the tensions that exist between organizational level talent management practices, the underlying talent philosophies and the individual career experiences of employees (Cascio and Aguinis, 2019; Rodrigues et al., 2020).

## 3 Research approach and methods

### 3.1 Research approach

The research problem is viewed through the lens of social constructivism, which states that reality is built through people’s perceptions and the meanings they ascribe to interactions and external phenomena (Williams, 2021). A generic qualitative approach was used in the research, to gain a detailed understanding of the ways in which talent management influences individual career experiences. In line with the underlying beliefs of subjectivism, this ensured access to meanings and a thorough understanding of how things happen in reality (Williams, 2021). This provided an understanding of the meanings and relationships between concepts, which was then used as the basis for developing a practical and theoretical contribution (Saunders et al., 2016; Alharahsheh and Pius, 2020).

### 3.2 Research method

The study was cross-sectional in nature, focusing on a single phenomenon at a single point in time. Non-probability, purposive sampling was used to select participants for data collection throughout the research process, which was conducted over two phases (Creswell and Poth, 2016; Lune and Berg, 2017).

During Phase 1, semi-structured interviews were used to collect data. The sample was drawn from a target population of talent management professionals (predominantly in South Africa) who are (1) currently working as practitioners in talent management and have insight into current and future talent requirements, and (2) represent various organizations. A small sample of 13 participants were chosen based on the inclusion criteria, as detailed in Table 2. During Phase 1 of the research, thematic analysis was used as the primary data analysis technique. This involved breaking data down into smaller units and elements and ascribing meaning to the data through a coding process (Lune and Berg, 2017).

**Table 2:** Overview of the interview participants according to inclusion criteria.

Participant code	Industry	Level of experience overall	Level of experience in talent management	Educational background
**Participant 1**	Financial services	Expert (10+ years)	Expert (10+ years)	Master’s degree, IOP, PhD candidate
**Participant 2**	Financial services/insurance	Expert (10+ years)	Advanced (8–10 years)	Master’s degree, PhD candidate
**Participant 3**	Transportation/financial services	Expert (10+ years)	Advanced (8–10 years)	Master’s degree, IOP
**Participant 4**	Airline	Expert (10+ years)	Expert (10+ years)	Master’s degree, IOP, PhD
**Participant 5**	Technology	Expert (10+ years)	Senior (5–8 years)	Master’s degree
**Participant 6**	Food delivery	Expert (10+ years)	Advanced (8–10 years)	Master’s degree, IOP
**Participant 7**	Mining/financial services	Expert (10+ years)	Senior (5–8 years)	Master’s degree
**Participant 8**	Logistics	Expert (10+ years)	Expert (10+ years)	Master’s degree
**Participant 9**	Transportation (government)	Expert (10+ years)	Advanced (8–10 years)	Master’s degree, IOP
**Participant 10**	Financial services	Expert (10+ years)	Advanced (8–10 years)	Master’s degree
**Participant 11**	Investments	Expert (10+ years)	Advanced (8–10 years)	Master’s degree
**Participant 12**	Healthcare	Expert (10+ years)	Advanced (8–10 years)	Honors degree
**Participant 13**	Financial services/banking	Expert (10+ years)	Expert (10+ years)	Master’s degree, IOP

IOP refers to professional registration as an Industrial and Organizational Psychologist with the Health Professions Council of South Africa.

Phase 2 of the research involved validation of the preliminary findings by an expert panel, through consensus agreement using the Delphi method. During this phase, the preliminary findings were presented to an expert panel through statements for validation. The expert panel included practitioners with deep expertise within talent management, as well as a theoretical understanding of how talent management is conceptualized within its broader context. Table 3 provides an overview of the statements presented to the expert panel. During this phase of the research talent management experts were carefully chosen to participate in the research. To ensure adequate validation of the findings through expert input, the Delphi method sample included eight participants, as detailed in Table 4. These participants were not among the practitioners who took part in the semi-structured interviews conducted during the first phase of the research.

**Table 3:** Overview of the statements presented to the expert panel for validation.

**Talent philosophy**
*Talent philosophy refers to the underlying beliefs about what talent is in the organization, the purpose that it serves, and the most effective ways to manage talent. Ultimately, philosophical beliefs drive the alignment of talent management practices in a coherent way.*
The underlying belief about who/what talent is should be aligned closely with the organizational reality.
The talent philosophy affects how talent is defined in the organization.
The talent philosophy determines the extent to which talent management practices are inclusive or exclusive in nature.
The talent philosophy should not reflect personal beliefs and preferences of what talent is and how it should be managed.
The three biggest organizational factors that affect the talent philosophy of the organization are the (1) organizational size, (2) current internal talent capacity, and (3) organization’s access to skills.
Talent can be considered as the inherent/innate attributes of people; therefore, they are managed with exclusivity (identify the existence of talents in a small group of individuals and manage differentially).
Talent can be considered as the attainable/developable attributes of people; therefore, they are managed with exclusivity (identify potential in a small group of individuals and manage differentially).
Talent can be considered as the inherent/innate attributes that all people possess; therefore, they are managed with inclusivity (align people with opportunities to display their talents).
Talent can be considered as the attainable/developable attributes that everyone can achieve; therefore, they are managed with inclusivity (everyone is talent).
The way in which talent is defined is largely dependent on the criticality and scarcity of the skills required in the organization.
Where the dominant skills required are highly scarce, talent may be defined as innate/ inherent that has to be sought.
Where the dominant skills required are less scarce, talent may be defined as attainable/ developable that can be built.
In larger organizations with more access to talent (internally or externally), a more exclusive approach to talent management is likely fit for purpose.
In smaller organizations with less access to talent (internally or externally), a more inclusive approach to talent management is likely fit for purpose.
The underlying talent philosophy determines the extent to which talent management practices are either self-directed or organizationally directed.

**Individual career factors**
*Individual career factors refer to the career structures and career experiences that affect talent management and which should be considered in talent management practices.*
Employees have more choice in the organizations and roles that they choose to experience their careers in.
Organizational purpose, employer brand and the organizational identity have an impact on the career choices that employees make.
Employees enter into organizations with a set of career expectations that may be implicit or explicit based on various organizational and individual factors.
The extent to which career expectations are met in the organization has a significant impact on the ability of the organization to retain talent.
Individual career experiences are largely the individual’s experience of the talent management practices in the organization.
Career experiences deliver on career expectations where it delivers on what the organization promises to deliver (explicit) and the psychological contract between the organization and employees.
The psychological contract has a critical role to play in managing the tension between organizational needs and individual career needs in talent management practices.
Transparency and trust are two of the most prominent mutual expectations between the organization and individuals, regardless of the type of psychological contract.
Talent management practices should reflect transparency and trust to deliver on mutual expectations.

**Table 4:** Overview of the expert panel participants according to inclusion criteria.

Participant code	Level of experience overall	Thought leadership/conceptual model development experience	Level of experience in talent management	Educational background
**Expert 1**	Expert (10+ years)	Expert (10+ years)	Expert (10+ years)	PhD, IOP
**Expert 2**	Expert (10+ years)	Expert (10+ years)	Expert (10+ years)	PhD, IOP
**Expert 3**	Expert (10+ years)	Advanced (8–10 years)	Advanced (8–10 years)	Master’s degree, IOP
**Expert 4**	Expert (10+ years)	Advanced (8–10 years)	Expert (10+ years)	Master’s degree, IOP
**Expert 5**	Expert (10+ years)	Advanced (8–10 years)	Expert (10+ years)	PhD, IOP
**Expert 6**	Expert (10+ years)	Advanced (8–10 years)	Expert (10+ years)	Master’s degree, IOP
**Expert 7**	Expert (10+ years)	Advanced (8–10 years)	Expert (10+ years)	Master’s degree, IOP
**Expert 8**	Expert (10+ years)	Advanced (8–10 years)	Expert (10+ years)	Master’s degree

IOP refers to professional registration as an Industrial and Organizational Psychologist with the Health Professions Council of South Africa.

Phase of the research consisted of two rounds of validation. The level of agreement was determined after each round, by listing the extent of agreement with each statement and considering additional insights shared by participants (Fink-Hafner et al., 2019). Although agreement between 55 and 70% is generally considered acceptable (Vernon, 2009), in the study, 80% agreement was used as the majority consensus threshold to ensure nuances and dynamics were incorporated into the model. Themes were narrowed down to an acceptable level of consensus through the input rounds. The themes and sub-themes identified during both these processes were integrated into the overall findings presented.

### 3.3 Strategies employed to ensure data quality and integrity

Several criteria were used to ensure the quality of the research. The comparability of the research to other research and contexts is referred to as transferability (Saunders et al., 2016; Nowell et al., 2017; Gray, 2019). This study ensured transferability by documenting the research process so that other researchers could conduct the study in different contexts and research settings. Second, dependability entails providing a trustworthy research account that others can understand and evaluate (Saunders et al., 2016; Gray, 2019). The careful documentation of the research process throughout its course enabled this. Third, credibility refers to how well the findings represent the data collected from participants (Saunders et al., 2016; Nowell et al., 2017). Sufficiency of data (gathering enough data to draw specific conclusions), triangulation of data (making connections between the literature and participant-collected data), member checking (testing data and interpretations with participants) during interviews, and validation through the Delphi method during data analysis all contributed to this. Finally, confirmability entails connecting data and interpretations in an understandable manner. It entails demonstrating how data is interpreted and conclusions drawn (Saunders et al., 2016; Nowell et al., 2017; Gray, 2019). Confirmability was achieved in the study through documentation and in-depth integration of findings and literature, as well as clear reasoning for theoretical, methodological, and analytical approaches throughout the study (Nowell et al., 2017).

## 4 Findings

The main findings across both phases of the research have been categorized into four broad themes, which are further expanded on within each section. The main themes are highlighted below:

- *Theme 1:* The impact of the philosophical beliefs around talent and talent management on individual careers
- *Theme 2:* Factors that impact the assumed talent philosophy and talent identification criteria
- *Theme 3:* The alignment between talent management practices and career enablement practices
- *Theme 4:* The role of the psychological contract and employee experience in managing mutual expectations

### 4.1 Theme 1: the impact of the philosophical beliefs around talent and talent management on individual careers

The first broad theme identified, relates to the impact of philosophical beliefs around talent management on careers. Specifically, this theme relates to how talent is defined in the organization (innate or acquired) and the extent to which the organization follows an exclusive or inclusive approach to managing talent.

Through participant interviews it is clear that there are inconsistencies in how talent is defined within organizations, as well as the extent to which talent management practices are inclusive or exclusive in nature. Despite these inconsistencies, participants recognized that this has an impact on individuals and their careers within the organization. Balancing talent management practices from the perspective of the organization with individual level careers was identified as a particular challenge for talent management practitioners, which was further validated by the expert panel during the second phase of the research. There was also disjointedness in how philosophical beliefs are translated into talent management execution, which poses further challenges for the career experiences of individuals.

*“So certainly, some talent we can develop. And then there’s definitely talent that we look for that is innate to people, particularly the soft skill side of things: leadership skills, management skills, relationship building skills, you know, those things are all things that, you know, more than opinion, I guess, in fact, I guess you can develop those things, but let us face it, some people are just better at dealing with customers and others, you know. So, we look at it from both perspectives.” – Participant 2, Financial Services/Insurance.*

*“… people bring certain strengths, experiences, skill sets, and that they can offer those up in the business. And it’s about finding the right the optimal place for those people in the business.” – Participant 6, Food Delivery.*

*“I think while everyone is considered talent, I think that in terms of the organization, you do really need to focus on those very, very critical positions.” – Participant 9, Transportation (Government).*

*“We believe everybody’s talent …” – Participant 5, Technology.*

*“We see each and every person working for us as talent, and each and every person wanting to work for us as talent.” – Participant 8, Logistics.*

*“I think the predominant view is that you can hire for potential.” – Participant 7, Mining/Financial Services.*

*“I think it’s detrimental in your business to put out exclusionary programs. And I think it has a negative impact, more so today than has in the past on, on your culture and on engagement. So, I do not I do not think it serves the business to have to have a very exclusive approach.” – Participant 6, Food Delivery.*

*“I think you will treat people the way you see them. And if you see some people as being less than others, you actually treat them in that way. So, for me, I think it was more around the cultural shift, are the cognitive shift that needs to take place, that actually will have resulted in how you see how you think about people, how you perceive them, and then ultimately, how you treat them.” – Participant 1, Financial Services.*

*“… and I think that the executive team were comfortable that from acquisition, if they feel that there is no fit, there would not be a lot of great people … so they are comfortable that it’s exclusionary to a point.” – Participant 11, Investments.*

During the expert panel validation, there was agreement that different approaches may hold value for the organization. Instead of defining a singular philosophical view, it is more important for organizations to be clear about their underpinning talent philosophy, how this impacts the talent experience and careers of individuals and how this should be translated into talent execution.

### 4.2 Theme 2: factors that impact the assumed talent philosophy and talent identification criteria

The results of the participant interviews showed that a variety of factors have an impact on the organization’s assumed talent philosophy as well as the criteria used for talent identification. Although these factors do not always clearly translate into a defined philosophy, they are seen to affect the underlying belief around who talent is and how talent should be managed implicitly or explicitly. Some of the views shared by participants are highlighted by the following quotes, to illustrate the diversity of views.

“And we said that as a business, we cannot afford certain leaders being at a higher level than other leaders. So as a business, our promise is that every single leader in our business will go through a leadership development program.” – Participant 3, Transportation/Financial Services.

“Development program or onboarding program allows, you know, the talent management team to have a pool of individuals who could potentially be ready for the future.” – Participant 4, Airline.

“That’s a really big conversation across our people domain at the moment is the skills-based talent practices, and what they mean for legacy talent practices.” – Participant 6, Food Delivery.

“The business still wants to function like a small business, even though it’s become complex. So, I think the realization that their talent model or their capability modeling, in spite of best efforts have actually not even matured.” – Participant 1, Financial Services.

“I think the mobility impact will start small and will grow over time. But it’s more going to be in terms of building connections across businesses and getting cross-functional exposure. And learning as opposed to necessarily being fully mobile from a permanent perspective.” – Participant 6, Food Delivery.

Table 5 provides a summary of all the factors that were identified during the participant interviews. These provide important context for understanding what needs to be considered in defining what is believed about talent, to create congruence and alignment with individual career expectations and experiences.

**Table 5:** Overview of the factors that affect talent philosophies.

Factors that affect the talent philosophy	How does it affect the talent philosophy?
Organization size, workforce composition, and strategic purpose	Practical considerations for managing talent. Speed of acquiring talent. Talent as a market differentiator. Feasibility of longer-term investments in talent.
Legacy/inherited beliefs in the organization	Dominant view of how talent is defined and managed. Overemphasis of past successes and failures. Reluctance to let go of tried and tested beliefs. Overreliance on personal beliefs.
Talent management maturity	Focus on how philosophy is operationalized through talent management practices and processes. Overreliance on personal beliefs about talent. Limited consideration of organizational context. Focus on transactional talent management practices.
Mobility and career management practices	Maturity and effectiveness of talent mobility and career management practices. Congruence between talent management and career experiences.
Adoption of employee experience/human-centered practice design	Consideration of the experience of employees in talent management. Congruence between philosophical talent management beliefs and career experiences in the organization.

During the expert panel validation, there was consensus that being identified as talent has an impact on the opportunities that are available to individuals. Moreso, it also impacts the extent to which individuals are in control of their career movements (for example being identified as a successor for a specific role). By understanding the factors that impact the talent philosophy of the organization, it is possible to align talent management execution and create clarity on mutual expectations as it relates to both employees and the organization.

### 4.3 Theme 3: the alignment between talent management practices and career enablement practices

Most interview participants view talent management practices and career management practices as separate practices within their organizations. This is largely due to the all-encompassing nature of talent management within organizations, which in most cases are aligned to the employee lifecycle. Much less attention is paid to career support, management or enablement as an organizational level practice, in comparison to talent management. During the participant interviews and expert validation process, talent management was however linked to broader development initiatives.

“And then we would have specific strategies to support your development as a high potential across the business.” – Participant 4, Airline.

“How do we then develop this person to get there?” – Participant 3, Transportation/ Financial Services.

“And that’s a very structured program over 2 years where we teach them how to fly a plane.” – Participant 4, Airline.

“And so there can be a place for everybody. And skills can be utilized at varying levels. And now all of a sudden, you do not need to run programs across your entire organization. If you can stimulate gigs and projects to tap into as part of a way of work, you start creating experiential learning opportunities at all levels of the business, across all functions.” – Participant 6, Food Delivery.

As summarized in Table 6, these linkages highlighted broad career development as an enabler of talent management. These development initiatives are however viewed as separate to talent management practices, which often results in disjointed efforts in practice.

**Table 6:** Links between talent management and development initiatives.

Development initiatives	Link to talent management
Individual development plans	Performance data is used as input to identify development gaps. Development based on targeted next role/ assignment. Used to track readiness for movement. Linked to individual career aspirations.
Mentoring and coaching	High-touch development for leaders and successors. Used to develop leadership capacity (by being a coach or mentor to others). Reverse mentoring for young talent pools. High-potential and high-performance development.
Organizational level development programs	Based on the future skills requirements and existing gaps in the organization. ‘Academies’ focused on critical and scarce skills to ensure a ready talent pool.
Leadership development programs	Leadership level talent development linked to succession pools. High-potential and high-performance development.
Self-paced learning (online and on-demand learning)	Self-driven development and self-identification of internal mobility opportunities. Talent data considered in talent identification or uncovering hidden skills.
Stretch assignments and projects	Supports internal mobility and develops institutional knowledge for critical skills.

### 4.4 Theme 4: the role of the psychological contract and employee experience in managing mutual expectations

Interview participants highlighted a number of changes in how employees experience their careers, as well as a change in expectations from the organization. The most notable factors are related to the need for visibility and transparency around what can be expected during the employment relationship, which impacts trust and ultimately the psychological contract. This includes workplace arrangements as well as career opportunities that are available. Further to this, the overall employee experience of talent at work seems to be more important than ever, which is seen as a mechanism for managing and delivering on expectations.

*“Our talent approach has become, in one sense, broader. So, we focus on leadership, and everyone needs to be leadership skills, we focus on making sure that we develop for levels not for roles, and we but then also, we create transparency in terms of saying, you can still choose where you want to go based on your skill set.” – Participant 2, Financial Services/Insurance.*

*“I think definitely a demand for more clarity and structure.” – Participant 6, Food Delivery.*

*“… And in the perfect world, you would probably assess the entire workforce, and then identify who your high potentials are, rather than, you know, just seeking the opinions of, of the line managers.” – Participant 4, Airline.*

*“They’re demanding a little bit of that structure and guidance to start building those career paths and not looking to be in those careers for as long. So, the skills then required … development pathways to help them get into different careers and play across the organization a lot more.” – Participant 6, Food Delivery.*

*“It’s about making sure we are listening to our people, understanding where our businesses are at, providing the right work environment, the correct working environment that is safe for people to flourish. But also, understanding that globally, everybody’s talking about remote working, and you know, what does that mean, for us?” – Participant 12, Healthcare.*

The expert panel validation further confirmed that the organization has to be clear about the role that they play in managing careers, as well as talent management decisions that impact careers.

## 5 Discussion

**Figure 1:**
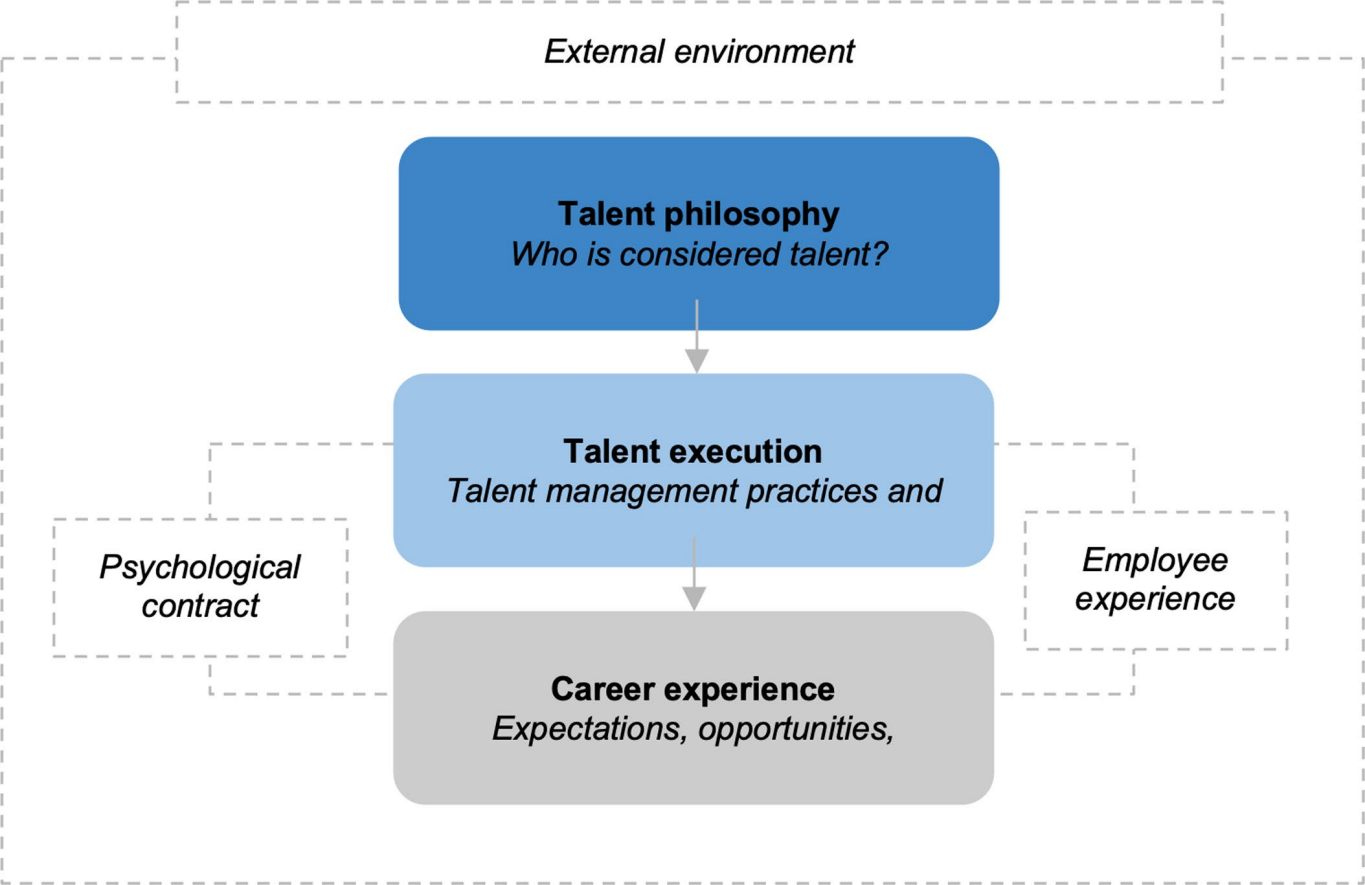
Overview of the impact of talent management execution on the career experience of talent execution.

Based on the findings of the study, there are a number of ways in which talent execution impacts the career experiences of talent. Aligned with the research question of this study, the interplays and impacts between these factors are outlined and visualized through Figure 1.

The study found that the philosophical beliefs that are held around talent management have a direct impact on talent management execution, through talent management practices. This means that the extent to which talent is believed to be innate or acquired and the extent to which talent management practices are inclusive or exclusive in nature, have an impact on individual career experiences. The execution of talent management practices has an impact on the career expectations that employees have, the access to and availability of career opportunities, as well as the relative career support and career choices available to individuals. Given the impact that talent management execution has on career experience, this tension has to be proactively managed to ensure that talent management unlocks value for both the organization and individuals. The study proposes that this tension can be managed by (1) clearly articulating the talent philosophy of the organization and making this visible through talent management execution, and (2) ensuring alignment between talent management practices and the career enablement offered to individuals to manage mutual expectations. The psychological contract as well as the lived experiences of individuals play an important role in managing the mutual expectations that exist between the organization and individuals. Each of these three components will be discussed in the following section.

### 5.1 The impact of a well-defined talent philosophy and its alignment with talent execution

As highlighted through the findings of the study, how talent is defined and decisions made around how talent is managed are two of the biggest factors that impact talent management execution. It steers the talent management practices that are prioritized in the organization as well as how careers are enabled within the organization (Meyers et al., 2019). The two biggest questions that have to be answered in defining the talent philosophy is (1) who is considered to be or have talent and (2) how should talent be managed (inclusive of all or exclusive to a few). There is no ideal talent philosophy that should be adopted by organizations, as each approach has its own benefits and drawbacks. The chosen talent philosophy has to be fit-for-purpose and reflect the reality of the organization. Each of these approaches, as well as the expected benefits and limitations, are discussed below as key considerations for the assumed talent philosophy of the organization.

When considering the talent philosophy detailed in Table 7, the focus of talent management practices should reflect processes for identifying talent internally with retention mechanisms in place that ensure talent is retained in the organization. Externally, a competitive approach may be required to attract and select the ‘best talent’ in the market. This has implications for the employer brand and attractiveness of the employee value proposition (EVP) in the market (Wiblen and McDonnell, 2020). Development in this context may be less focused on developing potential and rather focused on ensuring readiness for movement of talent into earmarked roles. It is likely that identification of talent could be based on performance or the display of talent in the past.

**Table 7:** Impact of innate and exclusive talent philosophy.

Talent Philosophy: Innate and Exclusive *Talent can be considered as the inherent/innate/stable attributes of people and are managed with exclusivity where identified*		
Practical application	Expected benefits	Possible pitfalls
Talent practices are focused on identifying the existence of talents in a small group of individuals and managing individuals differentially.	Organizations who outperform their competitors are organizations with access to more people with talent. A differentiated investment in talent is likely to show return on investment in the longer term as well-performing A-players are retained in the organization. These employees often show increased commitment to the organization as a result of the investments made in their development and careers, leading to reduced replacement costs.	Only a few employees benefit from this differentiated investment, and organizations run the risk of disengaging and alienating their broader workforce. It can also result in the inability of organizations to reach certain talent pools, particularly as the organizational needs change and evolve over time. The exclusive nature of this approach amplifies competition between organizations and vying for the same small pool of talent – this affects the sustainability of talent practices over time. Identifying talent through performance measures and intelligence measures may not be the best predictors of talent for future roles and ignores environmental factors and the influence of learning on future potential.

Adapted from Meyers et al. (2019).

When considering the talent philosophy detailed in Table 8, the focus of talent management practices should reflect processes that provide all employees with access to opportunities for mobility and development. Talent identification should focus on uncovering the talents of employees and providing platforms and opportunities to align individuals with opportunities to apply their talents (Wiblen and McDonnell, 2020; Aljbour et al., 2021). A greater focus on harnessing and utilizing talent in the business might be more fit for purpose. This includes visibility of available skills in the organization to make informed decisions about where these can be use. Development may be less focused on developing potential but rather on ensuring readiness for movement of talent, and the creation of fluid talent pools.

**Table 8:** Impact of innate and inclusive talent philosophy.

Talent philosophy: innate and inclusive *Talent can be considered as inherent/innate attributes that all people possess; therefore, they are managed with inclusivity*		
Practical application	Expected benefits	Possible pitfalls
Talent practices are focused on aligning people with opportunities to display their talents.	A positive, strengths-based approach to talent management adds value in its ability to identify strengths in individuals that may not have been uncovered. It allows for the development of strengths, and this is seen to lead to various positive individual outcomes (such as happy, driven and excited employees) and enhanced well-being, as well as better individual performance and increased organizational performance. In this approach, individual weaknesses are managed either through development programs or by partnering team members with complementary strengths and weaknesses to create diverse work teams. This approach leads to enhanced teamwork and team cohesiveness that cultivate appreciation for each team member’s contribution to the team.	This approach could create a fixed mindset about talents. Employees run the risk of believing that their traits and strengths are predetermined and cannot be changed, which could lead to avoidance of difficult tasks and could affect resilience. Despite leading to increased retention rates, organizations run the risk of not retaining the right employees who are considered to have tacit organizational knowledge that is more indispensable than other employees. This approach may also not fully allow organizations to compete for scarce or critical skills as there may not be enough of a differentiation to attract and retain these skills.

Adapted from Meyers et al. (2019).

In the talent philosophy detailed in Table 9, the focus of talent management practices should reflect processes that support the identification of potential and provide these employees with targeted opportunities for mobility and development. Identified talent should be retained through mechanisms that drive commitment to the organization in the longer term to deliver value (Wiblen and McDonnell, 2020; Aljbour et al., 2021). A clear definition of what is meant by potential should underpin the identification of talent, and resultant talent pools should be evaluated frequently to ensure they are considered and aligned with opportunities. Development in this context is focused on developing potential and ensuring readiness for movement of talent into earmarked roles.

**Table 9:** Impact of acquired and exclusive talent philosophy.

Talent philosophy: acquired and exclusive *Talent can be considered as attainable/developable attributes of people; therefore, they are managed with exclusivity where their potential is identified*		
Practical application	Expected benefits	Possible pitfalls
Identify potential in a small group of individuals and manage differentially.	Rather than viewing talent as a stable ‘have or have-not’ gift, this philosophy views talent as potential that can be cultivated to contribute to the organization. The benefit of providing development opportunities only to those who show potential is the expected return on investment for organizations. Often the designation of high potential in itself leads to better individual performance. Concepts such as talent transfer make the case for the transference of certain skills across various roles and occupations if certain prerequisites are met. This provides access to talent pools that may have remained out of reach for organizations.	The identification of potential remains a challenge. The exact combination of factors required to realize potential remains largely unknown. Potential required at a point in time might not be the potential required in future, and this is particularly relevant in the volatile and changing world of work. As a further result of the difficulty in identifying potential, the argument can be made that more employees actually have potential than an exclusive approach would highlight. This is particularly important when considering talent shortages as a theme in the 4IR, where talent could potentially also be developed or grown, where organizations are able to more accurately define and measure potential.

Adapted from Meyers et al. (2019).

In the talent philosophy outlined in Table 10, the focus of talent management practices should reflect processes that support the continuous growth and development of employees and align with their needs and aspirations (Meyers et al., 2019). Practically, consideration should be given to create visibility of opportunities as well as the skills and talents in the organization to align and deploy talent effectively. Instead of the identification of talent and directing efforts towards specific roles, a more skills-based approach and flexible talent pools may be more fit for purpose to align with this talent philosophy.

**Table 10:** Impact of acquired and inclusive talent philosophy.

Talent philosophy: acquired and inclusive *Talent can be considered as attainable/developable attributes that everyone can achieve; therefore, they are managed with inclusivity*		
Practical application	Expected benefits	Possible pitfalls
Everyone is considered as talent or can become talent.	The focus on continuous development and growth is expected to result in increased achievement and better performance. Managers are seen to be better coaches to their subordinates and can more accurately evaluate their performance as small incremental changes are highlighted. Highlighting that all employees can be extraordinary often leads to the Pygmalion effect or becomes a self-fulfilling prophecy. Developing a broad array of talents provides benefits to the organization in dealing with changing environments and volatility in the skills requirements of the organization.	Development of the entire workforce takes significant time, efforts and costs. It requires managerial support and other structures to support a conducive learning environment. Not all employees may want to or enjoy continuous development, resulting in deliberate interventions that spark motivation and encourage participation.

Adapted from Meyers et al. (2019).

**Figure 2:**
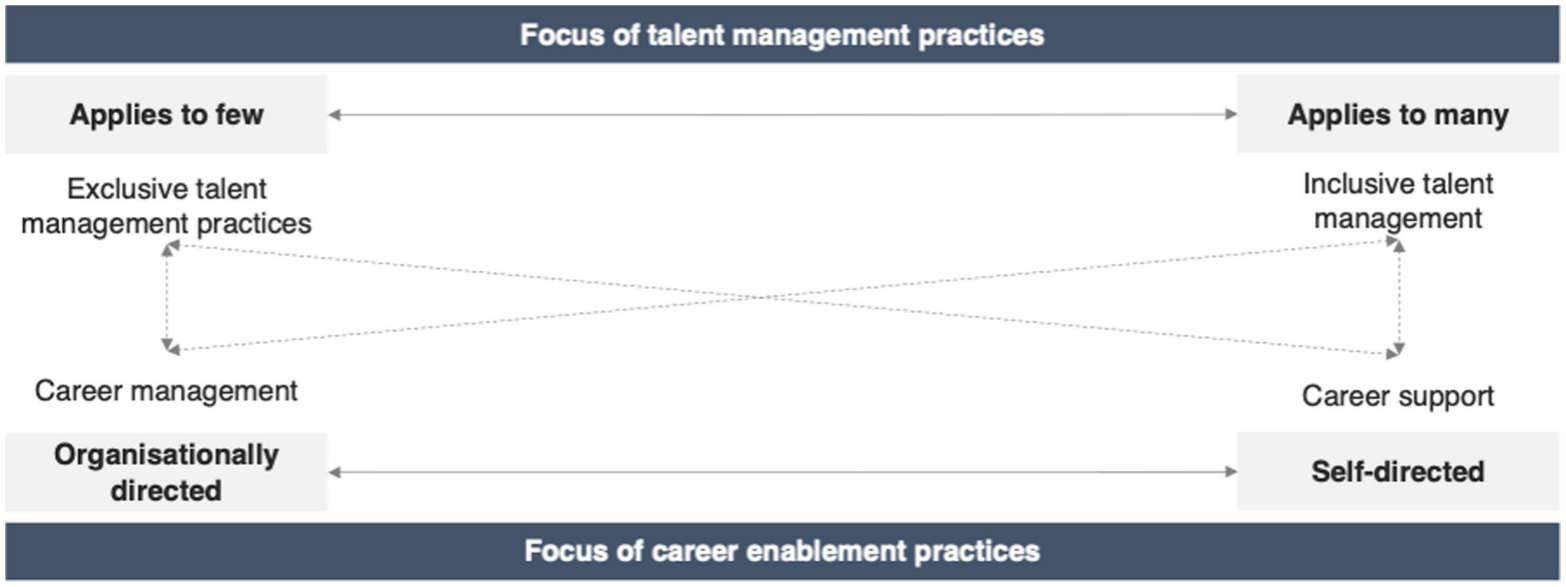
Interplay between talent management and career enablement practices aligned with the talent philosophy.

The interplay between talent management practices and career enablement practices in alignment with the talent philosophy of the organization, can be visualized by Figure 2.

Career enablement refers to the practices that organizations have in place to facilitate careers. This can be structured and managed through organizational practices, processes, and programs (less self-directed), or it can be less structured with a more self-directed approach. In whichever way career enablement plays out within the organization, it has to be considered in the context of the talent management practices, as these are interrelated and interdependent.

### 5.2 The alignment of talent management and career enablement practices to shape impactful career experiences

Based on the findings of the study, exclusivity in talent management approaches is more likely to lead to organizationally directed career management practices. This is a result of talent management decisions around identification and opportunities that are directed by the organization, and in turn impacts the type of career enablement that will support this talent management approach. It is, however, also possible for talent management practices to be exclusive in nature, but for the organization to drive and adopt more self-directed career enablement.

Similarly, inclusive practices naturally lean towards more broader career support, as opportunities are generally available to most or all and therefore cannot feasibly or effectively be directed by the organization through structured mechanisms. It is however also possible for the organization to apply inclusive talent management practices and provide structured career support to individuals.

**Figure 3:**
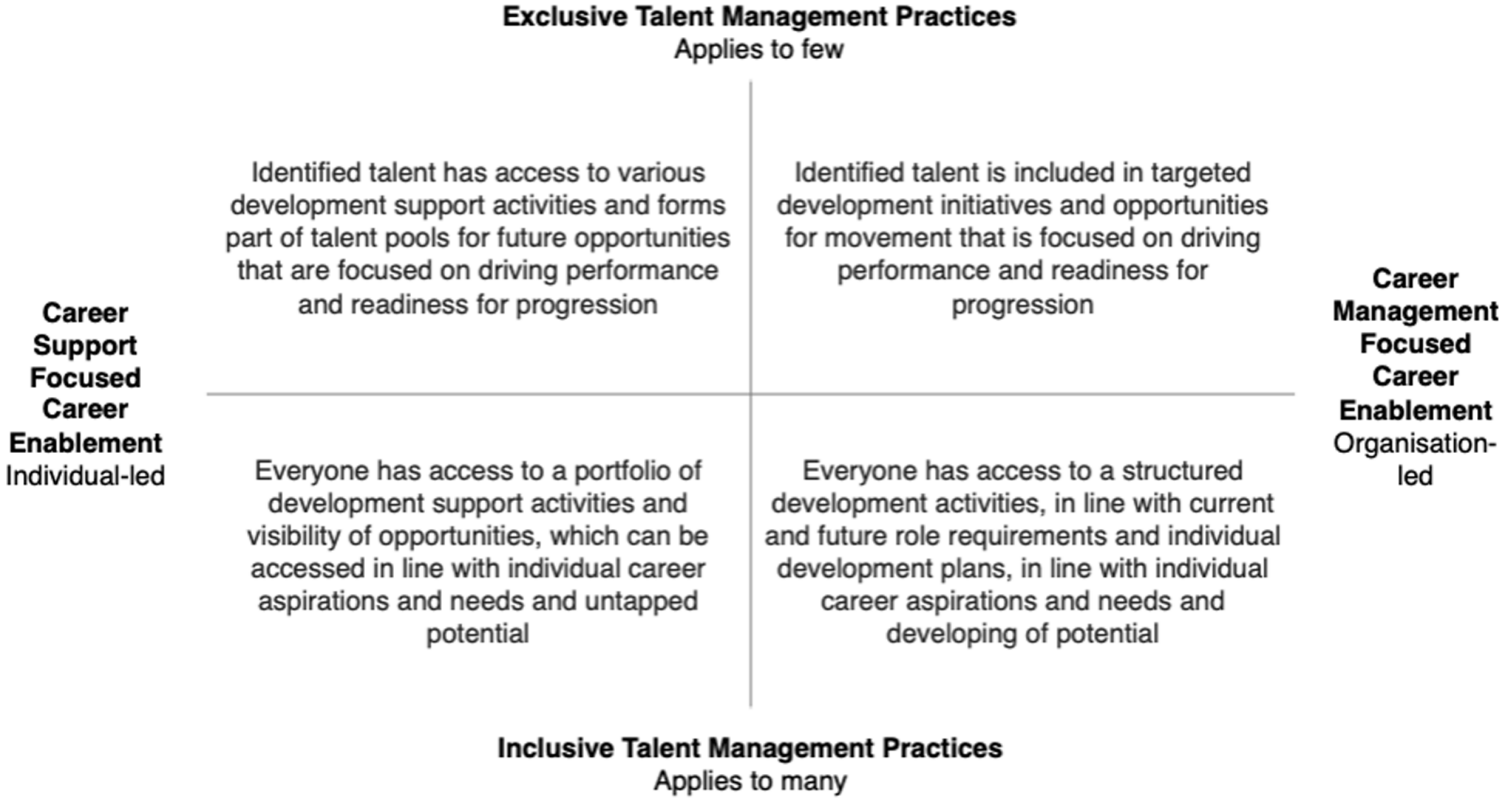
Alignment between talent management practices and career enablement practices, and associated practical implications.

Based on each of these possible scenarios, it is important to consider what this might look like practically to ensure that (a) approaches are fit for purpose, (b) is practical and feasible for the organization and (c) supports the management of the demand and supply of talent within the organization. Figure 3 provides an overview of the various possible alignments, as well as the practical implications of these approaches.

This alignment addresses three important career factors, which are seen to shape the career experiences of talent. These are career expectations, career choices and control. By explicitly defining what is believed about talent at a philosophical level, and translating that into talent management practices, mutual expectations are set. This means that individuals have clarity and transparency in how their careers are likely to be enabled within the organization, in the context of talent management. Related to this, it also defines the types of choices that individuals are likely to have within the organization. The nature of talent management execution may inhibit or promote career opportunities, options, and choices, which directly impact the career experiences of individuals. Lastly, the alignment between talent management and career enablement practices also signals the level of control individuals have over their careers. Here, consideration is given to the extent to which career enablement is individual-led or organization-led.

The more congruent talent management practices and career enablement practices are, the less risk the organization is likely to experience as a result of misaligned expectations and distrust. Alignment between talent management practices and career enablement has the potential to drive positive organizational outcomes, through the creation of shared value (Crowley-Henry et al., 2019).

### 5.3 The role of the psychological contract and lived experiences in the career experience

The psychological contract (regardless of the type that exists) plays a critical role in managing the tension between organizational needs and individual career needs, through clarity of expectations and aligned delivery (Perkins et al., 2021). Transparency and trust are two of the most prominent mutual expectations that exist between the organization and individuals, regardless of the type of psychological contract that exists. Talent management practices should reflect transparency and trust to deliver on mutual expectations. This is also referred to as the perceived individual-level of the talent climate or psychological climate with the organization by some researchers (King, 2017). The psychological contract demarcates the responsibility of the organization as well as the individual to manage mutual expectations with transparency and trust.

One of the best ways to create trust and transparency is through clear alignment of the talent management practices to the underlying talent philosophy, as well as consideration of the individual career experience impact of talent management practices. This also aligns to the signaling theory postulated by Spence (1973), which would indicate that inconsistency in talent management practices is likely to result in varying interpretations by individuals (King, 2017; Swailes, 2020). This means that mutual expectations are transparently communicated and addressed through the way that talent management is operationalized, as well as the experiences that this creates for talent. The psychological contract, as well as the employee experience, is therefore seen as all-encompassing or overarching considerations within the talent management model (King, 2017; Swailes, 2020).

Ultimately, the management and fulfillment of the psychological contract through lived experiences have a direct impact on the extent to which the organization is able to attract and retain talent within the organization and thus its longer-term ability to manage the supply and demand of talent (Perkins et al., 2021). The lived experience of talent is seen to reconcile the organizational talent management practices with their careers, making way for mutually beneficial talent management practices that impact organizational outcomes and sustainability in the longer term.

## 6 Theoretical and practical implications

The study provides a perspective on and consideration of individual careers in the context of talent management. The changing nature of careers and the role that organizations play in managing and enabling individual careers is critically important for the advancement of talent management theory (Wiernik and Kostal, 2019). Despite its relevance and importance, the impact of individual level career factors on talent management has not received sufficient attention in talent management literature (Crowley-Henry et al., 2019; Wiernik and Kostal, 2019); therefore, the study provides a unique contribution on how individual career choices, expectations and experiences are likely to affect talent management efforts in the organization. It makes the case for broader career enablement practices that are congruent with talent practices and unlock value for the organization. For talent management to be relevant, sustainable and successful in the changing world of work, it has to take the complexity of individual careers into account and reflect this reality in the operationalization and measurement of talent management practices (Crowley-Henry et al., 2019; Mensah, 2019), as demonstrated through the study.

## 7 Limitations and recommendations

Some limitations could not be addressed through the present study. The study focused on the ways in which talent management execution impacts career experiences of individuals, however there are a number of other factors that may also impact career experiences (Baruch and Rousseau, 2019; Kwon and Jang, 2022). It is recommended that other people practices, as well as organizational level practices which impact career opportunities, choice and control be further investigated to gather a more comprehensive understanding of the ways in which career experiences are shaped. These could include training and other formalized learning opportunities (Bozionelos et al., 2020), workload, organizational pace, and location (Straub et al., 2020), which are also seen to impact career sustainability.

## 8 Conclusion

This study investigated the ways in which talent management execution impacts on individual career experiences. The study also explored the ways in which talent management practices and career enablement practices can be aligned to unlock value for the organization as well as individuals. Employing a qualitative research design, the findings revealed the significant impact of talent management execution on career experiences of talent, which was found to be predominantly based on the impact on individual career opportunities, choice, and control. The study further highlights the importance of aligning career enablement practices to the organization’s talent philosophy, to deliver a congruent career experience. Lastly, the congruent lived career experience of talent is seen to lead to mutually beneficial talent management practices, that drives sustainable organizational impact in the longer term. This study contributes to the existing body of knowledge on careers and talent management, specifically focusing on the influence of talent management execution on careers, in the future world of work.

## Data availability statement

The datasets presented in this article are not readily available because the transcribed interviews contain identifiable data. Requests to access the datasets should be directed to marnavandermerwe@outlook.com.

## Ethics statement

The studies involving humans were approved by The University of Johannesburg research ethics committee of the Department of Industrial Psychology and People Management as part of a doctoral study under supervision of CH and PN. The studies were conducted in accordance with the local legislation and institutional requirements. The participants provided their written informed consent to participate in this study.

## Author contributions

MM: Conceptualization, Formal analysis, Investigation, Methodology, Project administration, Resources, Validation, Visualization, Writing – original draft, Writing – review & editing. PN: Conceptualization, Supervision, Writing – review & editing. CH: Conceptualization, Supervision, Writing – review & editing.
